# Older age is associated with a distinct and marked reduction of functionality of both alloreactive CD4+ and CD8+ T cells

**DOI:** 10.3389/fimmu.2024.1406716

**Published:** 2024-07-09

**Authors:** Nicolle H. R. Litjens, Amy C. J. van der List, Mariska Klepper, Derek Reijerkerk, Fréderique Prevoo, Michiel G. H. Betjes

**Affiliations:** Department of Internal Medicine, Division of Nephrology and Transplantation, Erasmus MC Transplant Institute, University Medical Center Rotterdam, Rotterdam, Netherlands

**Keywords:** age, donor-specific hypo-responsiveness, alloreactive, T cells, CD4/CD8 lymphocytes

## Abstract

**Introduction:**

Older recipient age is associated with a significant decreased risk for rejection after kidney transplantation which is incompletely understood.

**Methods:**

In a longitudinal study, circulating alloreactive T cells were assessed of young (≤45 years) and older (≥55 years) stable kidney transplant recipients. Alloreactive T-cells were identified by CD137-expression and phenotype, cytokine producing and proliferative capacity, were evaluated using multiparameter flowcytometry.

**Results:**

The results show that before transplantation frequencies of alloreactive CD4+ and CD8+ T-cells in older KT-recipients are significantly higher and shifted towards an effector memory-phenotype. However, the frequency of polyfunctional (≥2 pro-inflammatory cytokines) CD4+ T-cells was significantly lower and less IL2 was produced. The frequency of polyfunctional alloreactive CD4+ T-cells and proliferation of alloreactive T-cells donor-specifically declined after transplantation reaching a nadir at 12 months after transplantation, irrespective of age. A striking difference was observed for the proliferative response of alloreactive CD8+ T-cells. This was not only lower in older compared to younger recipients but could also not be restored by exogenous IL2 or IL15 in the majority of older recipients while the response to polyclonal stimulation was unaffected.

**Conclusion:**

In conclusion, older age is associated with a distinct and marked reduction of functionality of both alloreactive CD4+ and CD8+ T-cells.

## Introduction

Within the first year following kidney transplantation (KT), the incidence of T cell-mediated rejection (TCMR) is the highest but progressively declines over time (1–3). The reduced risk for TCMR has been linked to the decline in T-cell response to donor antigen post-transplantation, a process called donor-specific hyporesponsiveness (DSH) (4–6).

Recently, pre-transplant proportions of donor-reactive CD137+ memory CD4+ T cells expressing multiple cytokines (referred to as polyfunctional CD4+ T cells) were associated with a higher risk for acute T cell-mediated rejection (aTCMR) (7). The frequency of polyfunctional donor-reactive CD4+ T cells was shown to decline over time in stable KT recipients, which correlated with a reduced donor-specific proliferative capacity of CD4+ and CD8+ T cells (8). The progressive loss of these polyfunctional donor-reactive CD4+ T cells coincides with the decreased risk of aTCMR and could underlie the development of DSH.

Reduced TCMR risk has also been observed with aging. Most clinical studies on solid organ transplantation (including heart, kidney, and lung) indicate that increased recipient age (50 to >70 years) is associated with a reduced incidence of acute allograft rejection (9–13). The incidence of acute rejection in KT recipients decreases steadily with recipient age with rates 10% lower in patients above 65 years of age (2, 14, 15). Aging is associated with impaired T-cell function, leading to lower production of cytokines, impaired cytotoxic activity, and reduced proliferation in response to antigen stimulation (16). Aging also reduces thymic output as a result of thymic involution, leading to reduced numbers of naïve T cells and accumulation of memory T cells (16). However, very little is known about the effect of the recipient’s age on alloreactive T cells, and most studies have been performed in aged mice (16, 17).

The elderly have increased susceptibility to infections, reduced vaccination efficacy, and higher incidence of cancer and autoimmune diseases (18). In combination with immunosuppression, elderly KT recipients are at higher risk of these unwanted side effects (9, 18). Nevertheless, current dosing regimens of immunosuppression in kidney transplantation are based on a “one size fits all” approach. Therefore, it is important to understand how donor-reactive T cells are influenced by the recipient’s age to better determine rejection risk and improve dosing regimens. In addition, comparing age-dependent effects on donor-reactive T cells could reveal additional factors contributing to DSH.

We conducted a longitudinal study to investigate alloreactive T cells of both older (≥55 years) and young (≤45 years) stable KT recipients prior to and at 6, 12, and 36 months after transplantation. We used multiparameter flow cytometry to compare the frequency, differentiation status, cytokine production, and proliferation of alloreactive T cells.

## Materials and methods

### Study population

Older (≥55 years) and young (≤45 years) KT recipients were recruited from the Erasmus Medical Center and prospectively followed up for 3 years with blood sampling between August 2018 and May 2023. To minimize inter-assay variation, only complete series (i.e., prior to transplantation and 6, 12, and 36 months after transplantation) of recipient material were assayed within one experiment. The inclusion criteria for analysis were the absence of T-cell depletion therapy and stable graft function without evidence of recurrent disease and chronic rejection. For this study, heparinized peripheral blood samples at all designated timepoints from a total of 24 older recipients were present, with one participant being excluded due to chronic TCMR and one due to poor sample quality (**Table 1**). Similarly, samples from 25 young recipients were collected, but one participant was excluded due to having received T-cell depletion therapy and four due to poor sample quality (**Table 1**). All recipients received similar non-depleting induction therapy with basiliximab (Simulect®, Novartis Pharma, Amsterdam, the Netherlands). Maintenance therapy for the majority of patients consisted of tacrolimus (Prograf®, Astellas Pharma, Leiden, the Netherlands) and mycophenolate mofetil (MMF) (CellCept®, Roche, Woerden, the Netherlands). This study was approved by the Medical Ethical Committee of the Erasmus Medical Center, and all participating KT recipients gave written informed consent to participate in this study (MEC No. 2018–048). This study was conducted in accordance with the Declaration of Helsinki and the Declaration of Istanbul and in compliance with the International Conference on Harmonization Good Clinical Practice regulations.

**Table 1:** Patient demographics and transplant characteristics.

	Young	Older
Number of individuals	25	24
Age in years, median [IQR]*	39 [33–42]	63 [59–67]
Male, n (%)	15 (60%)	18 (75%)
Donor age in years, median [IQR]*	54 [40–61]	51 [39–57]
Donor male, n (%)	10 (40%)	13 (54%)
First kidney transplant, n (%)	23 (92%)	24 (100%)
Type of transplantation		
*Living donor, n (%)*	17 (68%)	20 (83%)
*Deceased donor, n (%)*	8 (32%)	4 (16%)
Number of mismatched HLA, median [IQR]		
*HLA class I*	3 [2,3]	2 [2,3]
*HLA class II*	1 [1,1]	1 [1,1]
Renal replacement therapy, n (%)		
*No (pre-emptive)*	6 (24%)	11 (46%)
*Dialysis*	17 (68%)	13 (54%)
*Prior transplant recipient*	2 (8%)	0 (0%)
Underlying kidney disease, n (%)		
*Nephrosclerosis/arteriosclerosis/hypertension*	6 (24%)	4 (17%)
*Primary glomerulopathy*	4 (16%)	4 (17%)
*Diabetic nephropathy*	3 (12%)	6 (25%)
*Polycystic kidney disease*	2 (8%)	4 (17%)
*Unknown*	10 (40%)	6 (25%)

IQR, interquartile range.

^*^At time of transplantation.

### PBMC isolation

Peripheral blood mononuclear cells (PBMCs) were isolated from heparinized peripheral blood samples on the day of blood sampling as described previously (19, 20) and stored until further use.

### CD3+ T-cell depletion of allogeneic stimuli

Stimulator PBMCs from a donor or a third-party control were depleted of CD3+ T cells (>98% depleted) using CD3 microbeads (Miltenyi Biotec, Bergisch Gladbach, Germany), according to the manufacturer’s instructions. Third-party stimulator cells had an equal number of, but different, Human leukocyte antigen (HLA) mismatches with the tested recipient as the donor. In **Supplementary Table 1**, the HLA typing for each kidney transplant recipient and source of stimulator cells (donor and third party) are listed. In addition, the assays for which the specific samples were used are listed as well including a reason for exclusion, when appropriate.

### Frequencies of cytokine-producing T cells within alloreactive T cells

The CD137 multiparameter flow cytometric assay was used to determine the frequencies of cytokine-producing cells as described previously (8). Following 15 hours of stimulation, cells were stained at the cell surface and intracellular as described before (8) using antibodies listed in **Supplementary Table 2**. Samples were measured on the Symphony A3 light (BD Biosciences, San Jose, CA, USA). During measurement, 0.5–1 million viable T cells were stored for subsequent analysis of frequencies of cytokine-producing cells and phenotypic characteristics using Kaluza version 2.1 software (Beckman Coulter, Woerden, the Netherlands). A representative example of a gating strategy for T-cell phenotype and cytokine expression is illustrated in **Supplementary Figure 1**. Allo-stimulated samples with poor-quality donor or third-party spleen cells (reflected by the absence of proliferation above the background when compared to the unstimulated condition) were excluded. This resulted in a total of N = 21 older and N = 19 young KT recipients analyzed for cytokine data.

### Proliferation assay

PBMCs of KT recipients were measured for proliferative capacity, as described previously (8, 21). Following 6 days of stimulation, supernatants were collected and stored until further use, cells were harvested, and extracellular staining was performed with the exclusion of CD137-directed antibody (**Supplementary Table 2**). Proportions of proliferating [carboxyfluorescein succinimidyl ester (CFSE)-negative] T cells were determined, and subsequent analysis was performed using Kaluza version 2.1 software (Beckman Coulter, Woerden, the Netherlands). A representative example of a gating strategy to identify proliferating T cells is included in **Supplementary Figure 2**. Allo-stimulated samples with poor-quality donor or third-party spleen cells (reflected by the absence of proliferation above the background when compared to the unstimulated condition) were excluded. This resulted in a total of N = 21 older and N = 20 young KT recipients analyzed for proliferation. In a separate set of experiments, the effect of exogenous IL2 on the proliferation of CD8+ T cells to alloantigen stimulation was explored. For this purpose, CD8+ T cells were isolated from CFSE-labeled PBMCs from seven young and 12 older KT recipients using the untouched CD8+ T-cell isolation kit (Miltenyi Biotec), according to the manufacturer’s instructions. Obtained CD8+ T-cell fractions had a purity above 94%. CD8+ T cells were stimulated with alloantigen (irradiated CD3-depleted PBMCs) in the absence and presence of increasing concentrations of human recombinant IL2 (Proleukin®, Clinigen Healthcare Ltd., Burton, UK). For a smaller group of KT recipients (three young and five older KT recipients), recombinant human IL15 (10 ng/mL; PeproTech EC Ltd., London, UK) was also added. In addition, isolated CD8+ T cells were stimulated with different concentrations ranging from 0.16 to 5 μg/mL of) plate-bound anti-CD3 (clone UCHT-1, BD). Following 6 days of stimulation, the proliferation of CD8+ T cells was measured via flow cytometry and analyzed as described above. Moreover, percentages of low- (IL2Rα), intermediate- (IL2Rβγ), and high-affinity (IL2Rαβγ) IL2 receptors were determined on day 0 and day 6 following staining of CD8+ T cells with antibodies directed to IL2Rα (CD25), IL2Rβ (CD122), and IL2Rγ (CD132) (**Supplementary Table 2**) and measured by flow cytometry.

### Cytometric bead array

After 6 days of proliferation in a mixed lymphocyte reaction, the concentration of IL2 in the supernatant was measured using the BD™ CBA Flex Set (BD, Erembodegem, Belgium) in conjunction with the BD CBA Human Soluble Protein Master Buffer Kit (BD), following the manufacturer’s instructions. Concentrations were calculated using linear regression analysis in GraphPad Prism 9.0.0 (GraphPad Software Inc., San Diego, CA, USA).

### Statistical analysis

Statistical analyses and graphs were created using GraphPad Prism 9.0.0 (GraphPad Software Inc., San Diego, CA, USA). Due to missing values, mixed-effects analysis instead of two-way ANOVA was used to compare the time course of cytokine-producing and proliferating T cells for young and older KT recipients. *Post-hoc* analyses were used to evaluate where significant effects were noted in time or between age groups. Appropriate paired and unpaired parametric tests were used to compare different timepoints within an age category and between different age categories per timepoint, respectively. Normally distributed data are depicted as the mean and standard error of the mean (SEM), and non-normally distributed data are depicted as the median and interquartile range (IQR). A p-value <0.05 indicates a statistically significant difference.

## Results

### Older KT recipients have increased frequencies of donor-reactive CD4+ and CD8+ T cells with a highly differentiated memory T-cell profile

**Figure 1:**
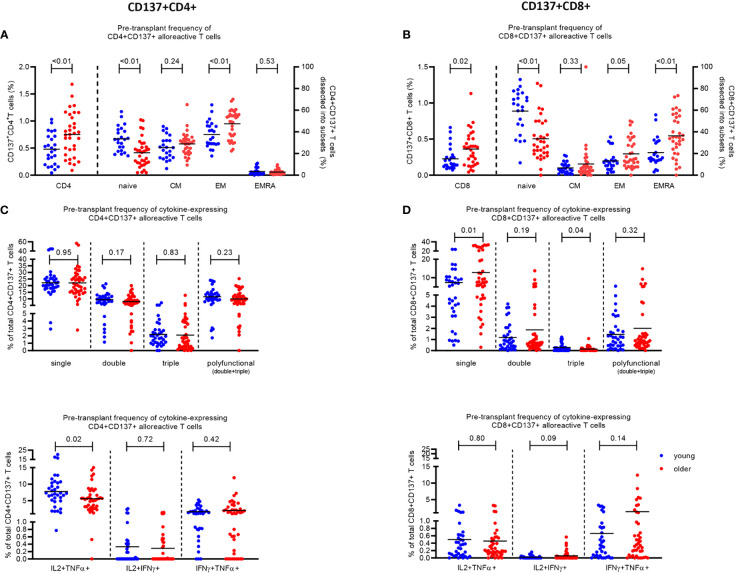
Phenotype and cytokine expression of alloreactive (CD137+) CD4+ and CD8+ T cells in young (≤45 years) and older (≥55 years) stable kidney transplant recipients pre-transplant. The pre-transplant percentage of **(A)** CD4+ and **(B)** CD8+ T cells expressing CD137 (left Y axis) and the percentage of CD137-expressing cells dissected into the different T-cell subsets (naïve, CM, EM, and EMRA) (right Y axis) within N = 25 young (depicted in blue) and N = 33 older (depicted in red) kidney transplant recipients following stimulation with alloantigen. Pre-transplant proportions of **(C)** CD4+ and **(D)** CD8+ T cells expressing one (single), two (double), three (triple), or at least two (polyfunctional) pro-inflammatory cytokines (IFNγ, IL2, and TNFα) in response to alloantigen (upper panel), as a percentage of CD137+ T cells, and the different combinations of double cytokine-producing cells (lower panel). The horizontal line represents the mean value. p-Values were calculated using unpaired t-test.

Prior to transplantation, older KT recipients had a higher percentage (0.75% ± 0.07%) of alloreactive CD137+ CD4+ T cells compared to younger KT recipients (0.48% ± 0.06%) (p < 0.01) (**Figure 1A**). The percentage of alloreactive CD137+ CD8+ T cells was also higher (0.36% ± 0.04%) in older KT recipients compared to 0.23% ± 0.03% in younger KT recipients (p = 0.02) (**Figure 1B**). Alloreactive CD4+ T cells were primarily of the memory phenotype in both young and older KT recipients. Of the alloreactive CD4+ T cells, young KT recipients had 25.5% ± 2.2% with a central memory (CM) and 37.6% ± 2.5% with an effector memory (EM) phenotype compared to 28.9% ± 1.8% CM and 47.5% ± 2.3% EM (p < 0.01) in older KT recipients (**Figure 1A**). Alloreactive CD8+ T cells were primarily of the naïve T-cell phenotype in young KT recipients where percentages amounted to 59.2% ± 4.1% compared to 33.6% ± 3.5% in older KT recipients (p < 0.01). In older KT recipients, the majority of alloreactive CD8+ T cells had a terminally differentiated effector memory (TEMRA) phenotype, which was significantly higher (p < 0.01) at percentages of 36.3% ± 3.4% compared to 20.9% ± 2.8% in younger KT recipients (**Figure 1B**).

### Older KT recipients have lower frequencies of CD4+ donor-reactive T cells expressing multiple pro-inflammatory cytokines pre-transplant

Although the total frequency of alloreactive CD4+ T cells was higher in older KT recipients, the percentage of alloreactive CD4+ T cells expressing pro-inflammatory cytokines (IFNγ, IL2, and TNFα) was lower compared to that in younger KT recipients prior to transplantation, reaching significance (p = 0.02) for the subset expressing IL2 and TNFα (IL2+TNFα+) (**Figure 1C**). The percentage of alloreactive CD4+ T cells expressing one, two, and three pro-inflammatory cytokines in older KT recipients amounted to 22.0% ± 1.6%, 7.9% ± 0.7%, and 2.1% ± 0.4%, respectively, compared to those in younger KT recipients with 22.2% ± 1.5% (p = 0.95), 9.3% ± 0.8% (p = 0.17), and 2.2% ± 0.3% (p = 0.83), respectively (**Figure 1C**, top). Older KT recipients had significantly lower frequencies (5.6% ± 0.6%) of alloreactive CD4+ T cells expressing both IL2 and TNFα compared to 7.8% ± 0.8% in younger KT recipients (p = 0.02) (**Figure 1C**, bottom). No significant difference in frequency of alloreactive CD8+ T cells expressing multiple cytokines was identified between age groups prior to transplantation apart from a lower frequency of triple cytokine-producing cells amounting to 0.14% ± 0.03% for older compared to 0.27% ± 0.06% for young KT recipients (p = 0.04) (**Figure 1D**).

### Polyfunctional CD4+ donor-reactive T cells decline post-transplantation, but lower frequencies are reached earlier in older KT recipients

Following transplantation, a significant effect of time on the frequency of donor-reactive CD4+ T cells expressing more than one pro-inflammatory cytokine was observed (p < 0.01) (**Table 2**). The effect of time on the frequency of polyfunctional donor-reactive CD4+ T cells varied across age groups with a significant interaction between time and age observed for donor-reactive CD4+ T cells expressing multiple pro-inflammatory cytokines including those expressing two (p = 0.04) and in particular the combination IL2+TNFα+ (p = 0.03) (**Table 2**). This finding indicates a more rapid decrease in polyfunctional donor-reactive CD4+ T cells in the older KT recipients.

**Table 2:** Influence of time and age on percentage of donor-reactive CD4 or CD8 T cells expressing two or more pro-inflammatory cytokines over time.

	Donor			Third-party		
	Time	Age	Time × Age	Time	Age	Time × Age
CD4+CD137+						
Single+	0.59	0.12	0.11	0.72	0.26	0.15
Double+	<0.01	0.85	0.04	0.44	0.70	0.53
IFNγ−IL2+TNFα+	<0.01	0.23	0.03	0.28	0.39	0.61
IFNγ+IL2+TNFα−	0.35	0.89	0.98	0.83	0.91	0.14
IFNγ+IL2−TNFα+	0.27	0.17	0.88	0.93	0.70	0.10
Triple+	<0.01	0.37	0.77	0.09	0.78	0.36
Polyfunctional	<0.01	0.98	0.06	0.29	0.70	0.38
CD8+CD137+						
Single+	0.52	0.03	0.91	0.42	0.04	0.59
Double+	0.35	0.37	0.49	0.58	0.33	0.56
IFNγ−IL2+TNFα+	0.09	0.91	0.57	0.66	0.53	0.68
IFNγ+IL2+TNFα−	0.47	0.42	0.54	0.12	0.78	0.52
IFNγ+IL2−TNFα+	0.72	0.31	0.58	0.71	0.22	0.69
Triple+	1.00	0.78	0.30	0.31	0.81	0.28
Polyfunctional	0.35	0.43	0.70	0.53	0.39	0.50

A mixed-effects analysis was performed to evaluate the effect of time after transplantation (four categories, i.e., prior to and M6, M12, and M36), age of the kidney transplant recipient at transplantation (two categories, i.e., being ≤45 years or ≥55 years), and the interaction of time and age on the parameter measured. p-Values are listed; p < 0.05 indicates a significant effect.

In general, no effect of time or age was observed for samples stimulated by third-party antigens as well as for frequencies of donor-reactive CD8+ T cells producing multiple pro-inflammatory cytokines (**Table 2**).

**Figure 2:**
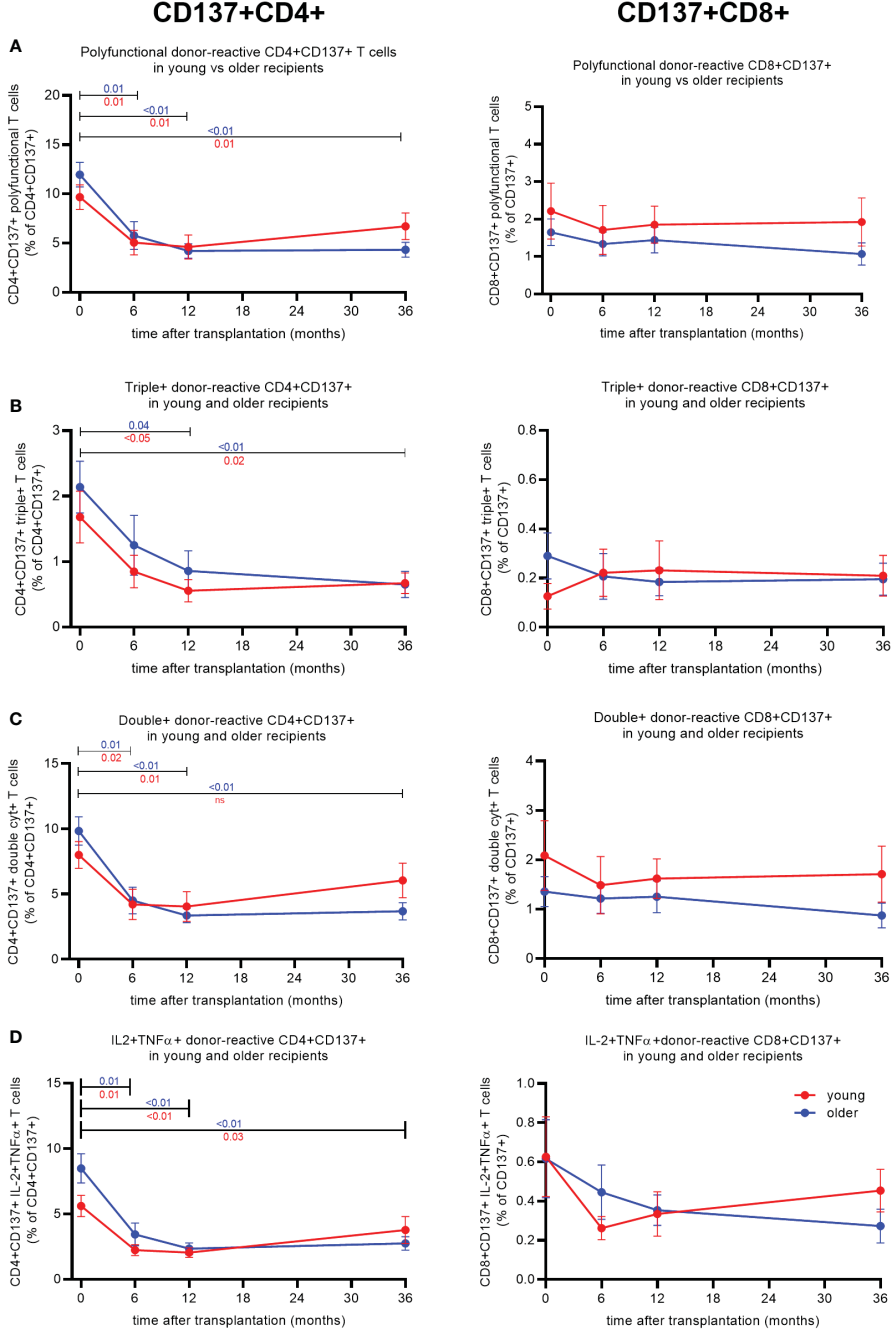
Cytokine expression of donor-reactive CD137+CD4+ and CD8+ T cells in young and older kidney transplant recipients post-transplant. Percentage of donor-reactive (CD137+)CD4+ (left) and CD8+ (right) T cells from pre-transplant and three timepoints post-transplant (6, 12, and 36 months) expressing two or more **(A)**, three **(B)**, two **(C)**, or the combination IL2 and TNFα **(D)**. Significant changes per age group over time were calculated using Tukey’s multiple comparisons test. A p-value of less than 0.05 is considered statistically significant. Data from young recipients are in blue, and those from older recipients are in red. Mean with standard error of the mean (SEM) depicted.

A significant decline in polyfunctional donor-reactive CD4+ T cells was observed 6 months post-transplant for both age groups (**Figure 2A**, left panel). The average ± SEM percentage of polyfunctional CD4+ T cells declined from 9.7% ± 1.3% to 5.1% ± 1.3% in older (p = 0.02) and from 12% ± 1.3% to 5.8% ± 1.4% in young (p < 0.01) KT recipients (**Figure 2A**, left panel). Following 6 months, the frequency of polyfunctional donor-reactive CD4+ T cells remained more or less at the same level for both age groups (**Figure 2A**, left panel). The observed decline was donor-specific, as third party-reactive CD4+ T cells did not decline over time (**Supplementary Figure 3A**).

Within the triple+ donor-reactive CD4+ T-cell population, a decline was observed for both age groups, reaching significance 12 months after transplantation. The proportion of triple+ declined from 1.7% ± 0.4% to 0.6% ± 0.2% in older (p < 0.05) and from 2.1% ± 0.4% to 0.9% ± 0.3% in young (p < 0.05) KT recipients (**Figure 2B**, left panel). The frequency of triple+ donor-reactive CD4+ T cells remained low 36 months post-transplant at 0.7% ± 0.2% (p = 0.02) and 0.7% ± 0.2% (p < 0.01) in older and young KT recipients, respectively (**Figure 2B**, left panel).

Proportions of double+ and IL2+TNFα+ donor-reactive CD4+ T cells of both young and older KT recipients declined significantly 6 months after transplantation (**Figures 2C, D**, left panel). Older KT recipients reached a lower frequency of IL2+TNFα+ donor-reactive CD4+ T cells at 6 months post-transplant declining from an average percentage pre-transplant of 5.6% ± 0.8% to 2.2% ± 0.4% (p < 0.01) compared to 8.5% ± 1.1% to 3.4% ± 0.9% in younger (p < 0.01) KT recipients (**Figure 2D**, left panel). Younger KT recipients reached this level after 12 months; i.e., the average proportion amounted to 2.4% ± 0.4% (**Figure 2D**, left panel).

No significant differences were observed for cytokine-producing donor-reactive CD8+ T cells (**Figures 2A–D**, right panel) for stable young and older kidney transplant recipients after transplantation.

### Older age is associated with decreased proliferation of alloreactive CD8+ T cells

**Figure 3:**
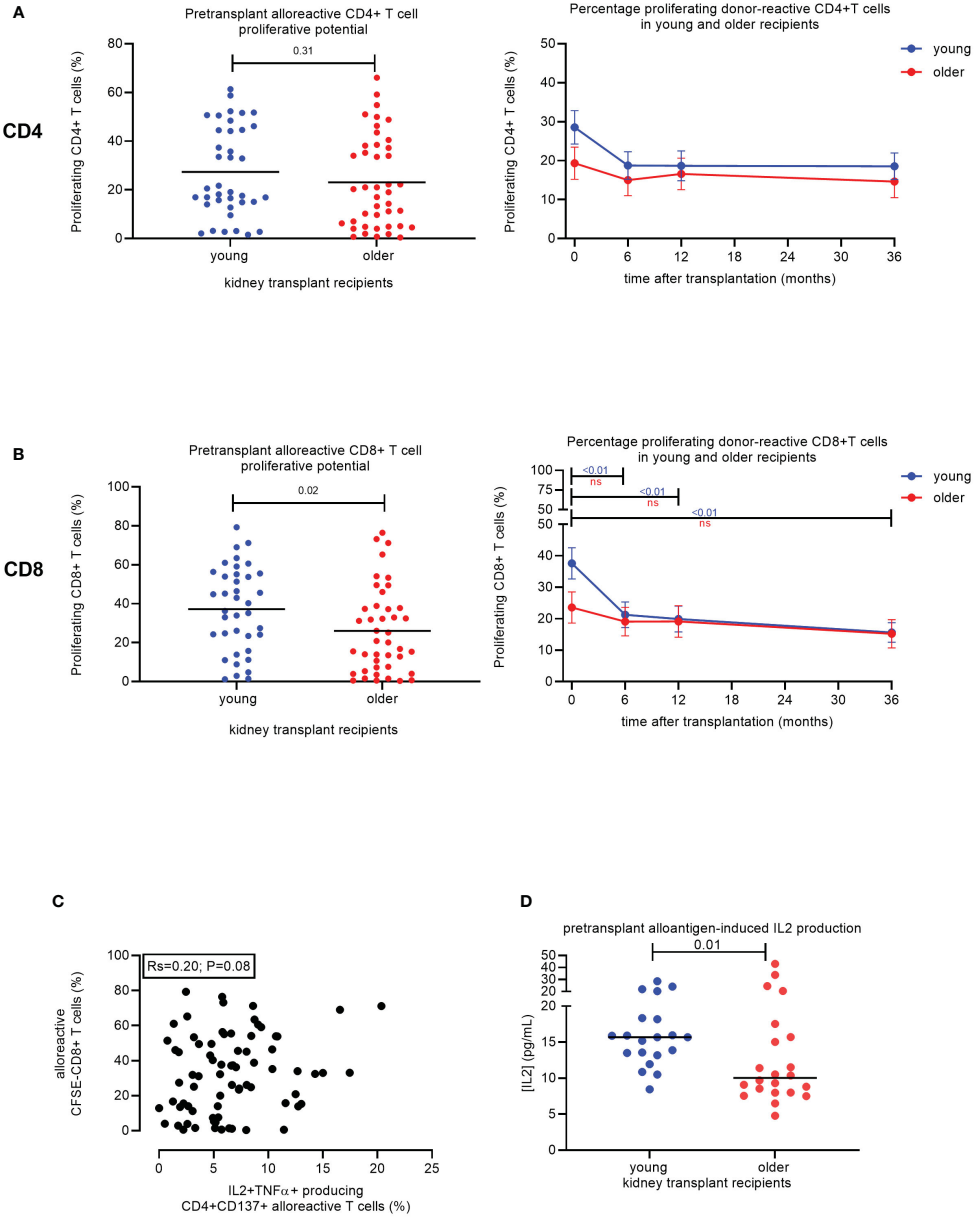
Proliferative capacity of alloreactive T cells in young versus older stable kidney transplant recipients. Pre-transplant percentage of proliferating (CFSE-negative) CD4+ T cells (top) and CD8+ T cells (bottom) of N = 38 and N = 42 alloantigen-stimulated samples from young and older recipients, respectively **(A)**. Percentage of proliferating CD4+ T cells (top) and total CD8+ T cells (bottom) to donor-antigen from pre-transplant and three timepoints post-transplant (6, 12, and 36 months) for N = 19 young (blue lines) and N = 21 older (red lines) kidney transplant recipients. p-Values were calculated using the unpaired t-test. A p-value of less than 0.05 is considered statistically significant. Mean with standard error of the mean (SEM) depicted **(B)**. Association between frequency of alloreactive CD137+CD4+ T cells expressing the combination of IL2 and TNFα (on the x-axis) with the proportion of proliferating CD8+ T cells following stimulation with alloantigen (on the y-axis). A total of N = 75 alloantigen-stimulated samples were included; N = 40 older and N = 35 young kidney transplant recipients. Pearson’s R and significance value are indicated **(C)**. The concentration of IL2 (pg/mL) measured in the supernatant after 6 days of proliferation of recipient PBMCs with alloantigen for young (N = 20) and older (N = 22) stable kidney transplant recipients pre-transplant is depicted **(D)**. CFSE, carboxyfluorescein succinimidyl ester; PBMCs, peripheral blood mononuclear cells. ns, not statistically significant.

Prior to transplantation, the percentage of proliferating alloreactive T cells in response to stimulation with alloantigen was significantly different between age groups for CD8+, but not CD4+, T cells (**Figure 3A**). Older KT recipients had a similar percentage of proliferating CD4+ T cells (23.1% ± 2.9%) compared to 27.4% ± 3.0% in young KT recipients (p = 0.31) and significantly fewer proliferating CD8+ T cells (26.0% ± 3.4%) compared to 37.3% ± 3.5% in younger KT recipients (p = 0.02) (**Figure 3A**). Of note, polyclonal stimulation of PBMCs with phytohemagglutinin (PHA) leads to substantial proliferation of CD8+ T cells, irrespective of age; i.e., average (SEM) % of proliferating CD8+ T cells amounted to 78.4% ± 3.9% versus 86.8% ± 3.0% (p = 0.09) for older and young KT recipients, respectively.

There was a significant effect of time after transplantation for the percentage of proliferating CD4+ T cells (p < 0.01) and CD8+ T cells (p < 0.01) (**Table 3**). In addition, a significant effect of the interaction between time and age (p < 0.01) was observed for the percentage of CD8+ T cells that proliferated in response to donor antigens (**Table 3**). No effect of time or age was observed for samples stimulated by third-party antigens (**Table 3**).

**Table 3:** Influence of time and age on percentage of proliferating (CFSE−) CD4 or CD8 T cells following donor stimulation over time.

	Donor			Third-party		
	Time	Age	Time × Age	Time	Age	Time × Age
**CFSE−CD4+**	<0.01	0.32	0.19	0.23	0.49	0.48
**CFSE−CD8+**	<0.01	0.38	<0.01	0.32	0.21	0.46

A mixed-effects analysis was performed to evaluate the effect of time after transplantation (four categories, i.e., prior to and M6, M12, and M36), age of the kidney transplant recipient at transplantation (two categories, i.e., being ≤45 years or ≥55 years), and the interaction of time and age on the parameter measured. p-Values are listed; p < 0.05 indicates a significant effect.

CFSE, carboxyfluorescein succinimidyl ester.

The average of proliferating CD4+ T cells declined in both age groups but remained fairly stable after 6 months post-transplant (**Figure 3B**, top). A similar but more pronounced pattern was seen for the CD8+ T cells. The average ± SEM of proliferating CD8+ T cells in older KT recipients declined to 19.1% ± 4.6% after 6 months and then decreased further to 15.2% ± 4.5% after 36 months (**Figure 3B**, bottom). Younger KT recipients had a significant decline in proliferating CD8+ T cells over time from 37.6% ± 5.0% at pre-transplant to 21.3% ± 4.1% at 6 months post-transplant (p < 0.01) (**Figure 3B**, bottom). One year after transplantation, the percentage of proliferating CD8+ T cells in young KT recipients declined further to 19.9% ± 4.2% and then to 15.7% ± 3.1% at 36 months post-transplant, reaching a similar level as older KT recipients (**Figure 3B**, bottom). No change in the percentage of proliferating T cells in response to third-party was observed over time for both age groups (**Supplementary Figure 3B**). Frequencies of alloreactive CD137+ CD4+ T cells capable of producing the combination of IL2 and TNFα tended to positively associate with the alloreactive proliferative potential of CD8+ T cells for KT transplant recipients (N = 75, Pearson’s R = 0.20, p = 0.08) (**Figure 3C**). However, older KT recipients (N = 22) had significantly lower concentrations of IL2 in cell culture supernatants. Values amounted to 10 pg/mL (IQR 8.0–16.2) compared to 15.7 pg/mL (IQR 13.3–18.3) (p = 0.01) for the younger group (N = 20) (**Figure 3D**).

### Age-related decrease of CD8+ T-cell proliferation cannot be restored by exogenous IL2

**Figure 4:**
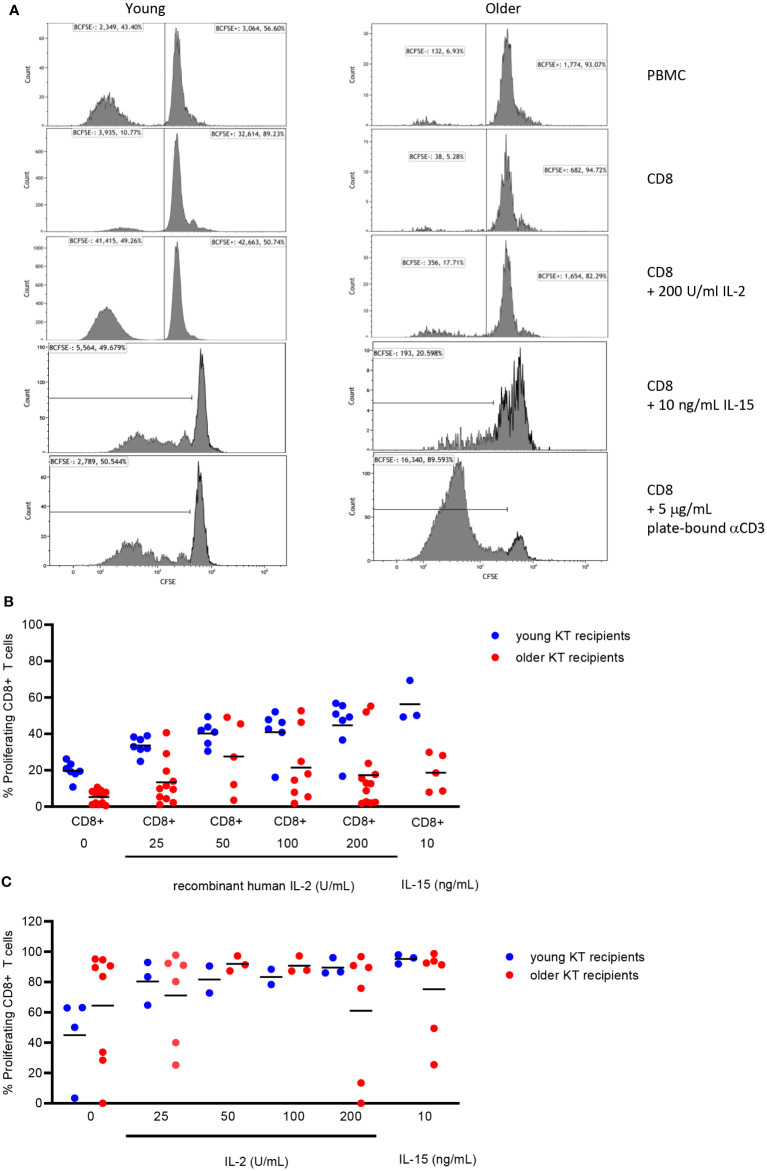
Proliferation of isolated CD8+ T cells from young and older kidney transplant recipients without/with addition of exogenous IL2 or IL15. Representative flow cytometric example of the percentage of alloantigen-induced proliferation (CFSE-negative) of CD8+ T cells within PBMCs (first row), isolated CD8+ T cells alone (second row) or supplemented with recombinant human 200 U/mL IL2 (third row), and 10 ng/mL recombinant human IL15 (fourth row) as well as the proliferative response of isolated CD8+ T cells to plate-bound anti-CD3 (5 μg/mL, fifth row) for young (left) and older (right) stable kidney transplant recipients **(A)**. Alloantigen-induced CD8+ T-cell proliferation to different doses of IL2 was measured in 19 kidney transplant recipients (seven young and 12 older), whereas that in response to IL15 was measured in eight kidney transplant recipients (three young and five older) **(B)**. In addition, CD8+ T-cell proliferation in response to plate-bound anti-CD3 (5 μg/mL) was measured for 12 kidney transplant recipients (four young and eight older) **(C)**. Blue circles indicate young kidney transplant recipients, and red circles indicate older kidney transplant recipients. Only pre-transplant PBMCs were used for more in-depth analysis of defective CD8+ T-cell proliferation. Horizontal line represents the mean. CFSE, carboxyfluorescein succinimidyl ester; PBMCs, peripheral blood mononuclear cells.

The results suggested that decreased IL2 production by alloreactive CD4+ T cells mediated the decreased proliferation of alloreactive CD8+ T cells in the older KT group. To test this hypothesis, CD8+ T cells were isolated from PBMCs of both young and older KT recipients prior to transplantation and evaluated the effect of increasing concentrations of exogenous IL2 on alloreactive CD8+ T-cell proliferation. First, the proliferation of isolated CD8+ T cells to an allogeneic stimulus was almost absent in the older KT recipients, and average ± SEM % amounted to 5.3% ± 1.1% as compared to 19.7% ± 1.8% in young KT recipients (p < 0.01). A dose–response effect was observed for increasing concentrations of IL2 added to alloantigen-stimulated CD8+ T cells in all young KT recipients but not in the majority of older KT recipients (**Figures 4A, B**). Only two out of 12 older KT recipients reached levels of the young KT recipients at 200 U/mL IL2, and average proliferation amounted to 17.3% ± 5.3% versus 44.7% ± 5.3% for the older versus young KT recipients, respectively. Basal expression of the low-, intermediate-, and high-affinity IL2 receptors was similar for young and elderly KT patients (**Supplementary Figure 4A**), but in the young exogenous IL2, it leads to induction of the high-affinity IL2 receptor (p < 0.01, **Supplementary Figure 4B**). Percentages amounted to 12.9% ± 1.1% versus 2.8% ± 1.6% for young and older KT recipients, respectively. Next, whether alloantigen-stimulated CD8+ T cells of older KT respond to IL15 was evaluated, as this cytokine shares both IL2Rβ and IL2Rγ with IL2 (**Figure 4B**). At 10 ng/mL, the average alloantigen-induced proliferation of CD8+ T cells of older KT recipients amounted to 18.6% ± 4.6% versus 56.3% ± 6.5% for younger KT recipients (p < 0.01). Strong proliferative CD8+ T-cell responses were observed after polyclonal stimulation with plate-bound anti-CD3, independent of age and the presence of exogenous IL2 or IL15 (**Figure 4C**). A dose–response curve of CD8+ T-cell stimulation with decreasing plate-bound anti-CD3 was performed to investigate whether the sensitivity of T-cell receptor (TCR) signaling differed for CD8+ T cells between young and old KT recipients. The results showed that proliferative response curves were practically overlapping, indicating a similar threshold for TCR-mediated induction of CD8+ T-cell proliferation (**Supplementary Figure 5**).

Given these results, exhaustion of CD8+ T cells of older KT recipients is not likely, and optimal stimulation of the TCR can overcome the lack of IL2 and IL15 responsiveness.

## Discussion

In this study, we observed age-related differences in the alloreactive potential of T cells of KT recipients. Older KT recipients had an increased proportion of alloreactive T cells and a higher representation of alloreactive T cells within the memory compartment. However, the pre-transplant frequency of polyfunctional alloreactive CD4+ T cells was significantly lower in older recipients, especially those capable of expressing both IL2 and TNFα. In addition, CD8+ T cells of older KT recipients had a significantly lower proliferative capacity in response to alloantigen at pre-transplant.

After transplantation, the frequency of polyfunctional donor-reactive CD4+ T cells with various combinations of pro-inflammatory cytokines declined significantly irrespective of the recipient’s age. Both young and older KT recipients had similarly low frequencies of polyfunctional donor-reactive CD4+ T cells at 1 year post-transplant, which remained low even at 3 years post-transplant. This decline is not related to T-cell exhaustion (22) but is likely caused by activation-induced cell death (8, 23). The proliferative capacity of CD8+ T cells of young KT recipients to donor-antigen declined significantly, reaching the same low level as observed for older KT recipients within 1 year post-transplant. In addition, the proliferation of alloreactive CD8+ T cells was markedly reduced in older KT recipients even in the presence of exogenous IL2 or IL15. These data suggest an intrinsic impairment of CD8+ T-cell proliferation and not simply a lack of CD4+ T-cell help. As CD8+ T cells proliferated adequately to a polyclonal stimulus, it seems likely that earlier events in CD8+ T-cell activation, i.e., signal 1 (TCR) and/or signal 2 (co-stimulation), are affected by age.

Data on T-cell aging and alloreactivity were derived from mouse models, and no human data on this subject were present. Adoptive transfer mouse models have revealed lower anti-donor Th1 responses including the reduced capacity of CD4+ memory T cells to produce IL2 in older mice (24–31). Also, T cells of young mice showed a significantly superior ability to produce IL2 and IFNγ after stimulation with young allogeneic dendritic cells compared to aged T cells (24). Transfer of T cells of older mice into younger hosts was associated with a delay in the tempo of fully MHC-mismatched skin allograft rejection (24, 27), and the proliferation of CD4+ and CD8+ memory T cells appeared to be lower in older mice (24, 25, 27, 32). Data on aged alloreactive CD8+ T cells are limited but indicate defective signaling pathways involving both IL2 and IFNγ (33, 34). The results obtained in this study are clearly in line with these animal experimental data on alloreactivity and in line with older studies on both humans and mice showing age-associated reduction of IL2 production (24, 35–37) and impaired proliferation of CD8+ (37, 38) and CD4+ (35) T cells.

The explanation for the age-related decrease in T-cell function may be found in altered cellular functions at different levels involved in TCR-mediated T-cell activation (39). In particular, defective TCR-mediated activation of T cells has been described for CD4+ T cells, which is associated with decreased phosphorylation of extracellular signal-regulated kinase (ERK) in aged T cells (40–42). In addition, loss of CD28, a characteristic of aging-related enhanced differentiation of T cells (17), was associated with a decreased risk for early acute T cell-mediated rejection (43).

However, the very poor alloreactive CD8+ T-cell proliferation in older recipients is a novel and unexpected finding. This phenomenon appeared independent of the cytokine-secreting profiles of these cells. Therefore, alloreactive CD8+ T-cell proliferation seems dissociated from the cytokine secretion profile, in contrast to the alloreactive CD4+ T cells (8). Older alloreactive CD8+ T cells were largely unresponsive to the known T-cell growth factors IL2 and IL15 and did not adequately upregulate the high-affinity IL2 receptor. Stimulation of the TCR using plate-bound anti-CD3 leads to vigorous T-cell proliferation, indicating that exhaustion or senescence is not a likely explanation. A more in-depth analysis of this phenomenon is warranted and may reveal features of aged alloreactive CD8+ T cells, which can lead to the development of biomarkers for aTCMR risk stratification and guide the lowering of immunosuppression.

Previous research in our laboratory has shown that increased pre-transplant frequencies of polyfunctional CD137+ donor-reactive T cells with an EM/terminally differentiated CD45RA+ effector memory (EMRA) profile are associated with early aTCMR (7). Therefore, the lower frequencies of polyfunctional alloreactive CD4+ T cells in older KT recipients observed prior to transplantation in this study likely contribute to a lowered risk for aTCMR early after transplantation.

The generation of CD8+-mediated cell toxicity requires help from CD4+ T cells via activation of the antigen-presenting cell (44). This can only be partly attributed to IL2 production by CD4+ T cells, which is critical for antigen-driven clonal expansion of activated T cells (45). The lower frequency of donor-reactive CD4+ T cells producing IL2 and the IL2-independent reduced proliferative potential of CD8+ T results in a dual reduction in alloreactivity to donor antigen in older KT recipients. The timeline of the declining incidence of aTCMR matches the observed decline in donor-reactive polyfunctional CD4+ T cells. At longer follow-up, the frequency of alloreactive CD4+ and CD8+ T cells and the proliferative response in a mixed lymphocyte reaction (MLR) reached a similar low level for both young and older KT recipients. This may explain why older KT recipients have a lower risk for aTCMR early after transplantation compared to younger KT recipients but that aTCMR incidence for all, irrespective of age, is uncommon between 1 and 5 years, rare after 5 years, and virtually absent beyond 10 years (2, 46).

Limitations of the study involved the amount of recipient PBMCs, which were prospectively collected at different timepoints after transplantation. The amount of PBMCs was not always sufficient to perform the different experiments. However, we aimed to include a complete time course for a recipient studied. Another limitation was the quality of spleen cells as the source of donor (or third-party) cells for recipients receiving a kidney from a deceased donor. As spleens were not always processed within 24 hours, their use as stimulator cells was hampered, leading to the exclusion of a recipient upon performing the experiment. In addition, we were only able to measure direct alloimmune responses and cannot conclude what happens to indirect anti-donor reactive T cells. Further research is warranted; however, no *in vitro* assays are available for studying solely indirect alloimmunity. Including longer time intervals after transplantation allows for more definite conclusions to be drawn with respect to age-related differences in alloreactive T-cell immunity. In this respect, an earlier cross-sectional study including stable kidney transplant recipients more than 20 years after transplantation revealed that donor-reactive T cells remain low and even become undetectable (i.e., reaching levels above background) (8). In conclusion, this study found significant age-related alterations in the functionality of alloreactive CD4+ and CD8+ T cells with a strikingly low proliferation of alloreactive CD8+ T cells in the older recipients. After transplantation, a time-dependent loss of donor-specific T-cell alloreactivity leads to a similar profile of donor-specific hyporesponsiveness. Studies tapering immunosuppressive medication are warranted to assess the role of these parameters in identifying kidney transplant recipients that can be safely reduced in immunosuppressive medication.

## Data availability statement

The original contributions presented in the study are included in the article/**Supplementary Material**. Further inquiries can be directed to the corresponding author.

## Ethics statement

The studies involving humans were approved by Medical Ethical Committee of the Erasmus Medical Center (MEC No. 2018-048). The studies were conducted in accordance with the local legislation and institutional requirements. The participants provided their written informed consent to participate in this study.

## Author contributions

NL: Conceptualization, Data curation, Formal analysis, Funding acquisition, Investigation, Methodology, Supervision, Visualization, Writing – original draft, Writing – review & editing. AV: Data curation, Formal analysis, Investigation, Methodology, Visualization, Writing – original draft, Writing – review & editing. MK: Data curation, Formal analysis, Investigation, Methodology, Writing – review & editing. DR: Investigation, Methodology, Writing – review & editing. FP: Investigation, Methodology, Writing – review & editing. MB: Conceptualization, Funding acquisition, Investigation, Project administration, Resources, Supervision, Writing – original draft, Writing – review & editing.
