# Supplementary material for: Older age is associated with a distinct and marked reduction of functionality of both alloreactive CD4+ and CD8+ T cells
Source: Front Immunol. 2024 Jul 9;15:1406716. doi: 10.3389/fimmu.2024.1406716 (PMC11263037; doi:10.3389/fimmu.2024.1406716)
Supplement: Supplementary file 1 [file DataSheet_1.pdf]

Supplementary Material

Supplementary Table 1 HLA typing for kidney transplant recipients and source of stimulator cells and their use in assays

				assays		
**Recipients**	**HLA type recipient (A:B:DR)**	**HLA type donor (A:B:DR)**	**HLA type third party (A:B:DR)**	**cytokine producing cells**	**proliferation**	**reason for exclusion**
O1	A1 B8 B35 DR1 DR5 DR11	A1 A19 A30 B13 B35 DR5 DR11 DR7	A3 B15 B62 B35 DR1 DR6 DR13	yes	yes	
O2	A1 A31 B27 B51 DR7 DR13	A2 A29 B44 DR7 DR1	A10 A34 A28 A68 B5 B52 B17 B58 DR5 DR11 DR6 DR13	yes	yes	
O3	A2 A11 B7 B35 DR7 DR15	A29 B44 DR1 DR7	A2 A3 B5 B51 B35 DR2 DR16 DR3 DR17	yes	yes	
O4	A31 A32 B39 B51 DR13	A2 A31 B39 B44 DR7 DR13	A9 A24 A19 A33 B5 B51 B17 B57 DR2 DR15	yes	yes	
O5	A2 A10 A26 B15 B62 B40 B61 DR6 DR14 DR7	A1 A26 B38 B40 DR8 DR13	A2 A9 A23 B15 B62 B21 B49 DR4 DR5 DR11	yes	yes	
O6	A2 A11 B15 B18 DR15 DR16	A2 A24 B15 DR4	A2 A19 A32 B15 B62 DR2 DR15 DR5 DR12 DR51	yes	yes	
O7	A1 A2 B17 B57 B37 DR4 DR7	A2 B17 B57 B18 DR5 DR11 DR7	A1 B35 B37 DR1 DR4	yes	yes	
O8	A3 A68 B35 B44 DR13 DR14	A2 A68 B44 B57 DR7 DR14	A1 A3 B15 B63 B35 DR2 DR15 DR6 DR13	yes	yes	
O9	A2 A19 A32 B12 B44 B22 B56 DR4 DR5 DR11	A2 A31 B15 B40 DR4	A9 A24 B5 B51 B35 DR5 DR12	yes	yes	
O10	A2 A9 A24 B5 B51 B15 B62 DR4 DR6 DR14	A24 A32 B15 B35 DR4 DR13	A2 A11 B17 B57 B40 B60 DR4 DR7	yes	no	too few cells for proliferation
O11	A1 A11 B8 B35 DR3 DR17 DR4	A1 A9 A23 B8 B21 B49 DR2 DR15 DR3 DR17	A2 A3 B18 B27 DR2 DR15 DR5 DR11	yes	yes	
O12	A1 A19 A33 B8 B15 B63 DR3 DR17	A1 A19 A32 B8 B15 B62 DR2 DR15 DR3 DR17	A1 B8 B15 B62 DR3 DR17 DR4	no 3P/yes don	no 3P/yes don	bad quality stimulator cells
O13	A2 A29 B52 B53 DR9 DR13	A2 B5 B51 B16 B38 DR2 DR15 DR9	A2 B12 B44 B40 B61 DR4 DR6 DR13	yes	yes	
O14	A3 A19 A33 B14 B65 B35 DR1 DR8	A11 B17 B57 B35 DR1 DR6 DR14	A3 A9 A24 B7 B14 B65 DR1 DR2 DR15	yes	yes	
O15	A2 A28 A68 B35 DR2 DR16 DR6 DR13	A2 A28 A68 B12 B44 B15 B62 DR6 DR13	A2 A28 A68 B5 B51 B7 DR2 DR15 DR4	no don/ yes 3P	no don/ yes 3P	bad quality stimulator cells
O16	A3 A19 A32 B12 B44 B14 B65 DR5 DR12 DR6 DR13	A24 A9 A33 A19 B44 B12 B35 DR15 DR2 DR7	A1 A10 A26 B22 B55 B27 DR2 DR16 DR5 DR11	yes	yes	
O17	A9 A24 A11 B18 B40 B61 DR2 DR15 DR4	A3 A26 B7 B40 B60 DR4 DR15 DR5	A1 A2 B15 B62 B35 DR4	no	no	recipient diagnosed with chronic rejection
O18	A1 A2 B8 B15 B62 DR3 DR17 DR6 DR13	A3 B35 B37 DR1 DR4	A2 A19 A33 B70 B71 B17 B57 DR7 DR8	yes	yes	
O19	A2 A68 A28 B7 B60 B40 DR1 DR13 DR6	A3 A11 B8 B63 B15 DR17 DR3 DR13 DR6	A9 A23 A19 A33 B12 B45 B17 B58 DR6 DR13 DR8	yes	yes	
O20	A1 A10 A26 B17 B58 B21 B50 DR3 DR17 DR10	A3 A19 A32 B8 B12 B44 DR7 DR8	A2 A9 A23 B21 B49 B40 B60 DR2 DR15 DR6 DR14	yes	yes	
O21	A9 A24 B7 B16 B39 DR2 DR15 DR4	A2 A9 A24 B7 B15 B62 DR2 DR15 DR6 DR14	A1 A9 A24 B35 DR2 DR15 DR5 DR11	yes	yes	
O22	A1 A28 A68 B17 B57 B35 DR1 DR7	A1 A3 B39 B57 DR1 DR8	A1 A9 A24 B5 B51 B35 DR1 DR2 DR16	yes	yes	
O23	A1 B7 B8 DR15 DR2 DR17 DR3	A3 A26 A10 B27 B35 DR15 DR2 DR7	A11 A19 A33 B12 B44 B18 DR2 DR15 DR5 DR11	no	yes	intracellular staining not okay
O25	A9 A24 A19 A33 B14 B65 B35DR1 DR5 DR11	A24 A68 B7 B35 DR11 DR15	A3 A19 A29 B14 B65 B40 B60 DR1 DR4	yes	yes	
Y1	A9 A24 B5 B51 B17 B57 DR1 DR5 DR11	A1 A24 B08 B49 DR1 DR3	A2 B7 B12 B44 DR2 DR15 DR5 DR11	yes	yes	
Y2	A2 A3 B27 B35 DR8 DR16	A1 A24 B18 B37 DR11 DR13	A11 A19 A33 B5 B51 B12 B44 DR7 DR10	yes	yes	
Y3	A3 A24 B15 DR13	A32 A68 B35 B47 DR4 DR15	A1 A2 B8 B27 DR1 DR3 DR17	yes	yes	
Y4	A2 A19 A32 B7 B40 B60 DR2 DR15 DR6 DR13	A1 A2 B8 B40 DR4 DR13	A19 A32 A28 A68 B7 B37 DR2 DR15 DR6 DR13	yes	yes	
Y5	A11 B15 B40 DR4 DR9	A11 B13 B40 DR9 DR15	A11 B5 B52 DR2 DR15 DR4	yes	yes	
Y6	A2 A19 A30 B17 B57 B58 DR4 DR9	A2 A10 A26 B5 B51 B40 B61 DR4 DR6 DR13	A1 A19 A30 B8 B12 B44 DR3 DR17 DR9	no	no	bad quality responder/stimulator cells
Y7	A1 A11 B17 B57 B27 DR7 DR9	A3 B13 B18 DR7 DR9	A1 A9 A23 B7 B12 B44 DR7	no	no	bad quality responder/stimulator cells
Y8	A1 A28 A68 B5 B51 B8 DR4	A2 A28 A68 B5 B51 B7 DR2 DR15 DR4 D	A1 A3 B8 B12 B45DR3 DR17 DR4	no	no	bad quality responder/stimulator cells
Y9	A1 A2 B17 B40 DR15 DR7	A2 A9 B17 B40 B57 DR5 DR7 DR12	A2 A3 B7 DR2 DR15 DR4	no	no	bad quality responder/stimulator cells
Y10	A1 A9 A24 B7 B8 DR2 DR15 DR3 DR17	A2 A24 A9 B7 B39 B16 DR15 DR2 DR8	A1 A3 B8 B37 DR3 DR17 DR10	yes	yes	
Y11	A11 B7 B40 B60 DR2 DR15 DR3 DR17	A24 A9 A68 A28 B35 B60 B40 DR15 DR2 DR4	A1 A3 B7 B17 B57 DR3 DR17 DR7	yes	yes	
Y12	A2 A10 A26 B7 B15 B62 DR4 DR5 DR11	A1 A2 B8 B50 B21 DR17 DR3 DR7	A3 A10 A26 B16 B38 B37 DR6 DR13 DR10	yes	yes	
Y13	A2 A9 A24 B15 B62 B21 B50 DR6 DR13 DR8	A3 A28 A68 B12 B44 B16 B39 DR2 DR15 DR16	A1 A19 A32 B8 B17 B58 DR3 DR17 DR6 DR14	yes	yes	
Y14	A9 A24 A19 A30 B5 B52 B42 DR4 DR10	A29 A30 A19 B58 B17 B42 DR13 DR6 DR10	A9 A24 A11 B5 B51 B16 B39 DR1 DR4	yes	yes	
Y15	A11 A19 A32 B21 B50 B35 DR1 DR3 DR17	A11 A19 A32 B12 B44 B35 DR1 DR5 DR11	A11 A11 B17 B57 B35 DR1 DR6 DR14	yes	yes	
Y16	A2 B7 B17 B57 DR2 DR15 DR7	A2 A11 B7 B17 B57 DR2 DR15 DR7	A2 A9 A24 B7 B17 B57 DR2 DR15 DR7	yes	yes	
Y17	A9 A24 A11 B15 B62 B35 DR1 DR4	A2 A19 A30 B13 B35 DR6 DR13 DR7	A1 A10 A26 B22 B55 B27 DR2 DR16 DR5 DR11	yes	yes	
Y18	A19 A29 A32 B12 B44 DR1 DR7	A3 A29 A19 B44 B12 B27 DR15 DR2 DR7	A1 A19 A29 B8 B12 B44 DR7 DR8	yes	yes	
Y19	A24 A9 A33 A19 B58 B17 B18 DR4 DR11 DR5	A1 A24 A9 B51 B5 B37 DR17 DR3 DR4	A2 A9 A24 B12 B44 B40 B60 DR5 DR12 DR8	no	yes	intracellular staining not okay
Y20	A1 A2 B15 B62 B40 B60 DR1 DR3 DR17	A2 B51 B5 B8 DR17 DR3 DR11 DR5	A1 B35 B37 DR1 DR4	yes	yes	
Y21	A11 A19 A30 B5 B52 B13 DR2 DR15	A1 A19 A30 B7 B13 DR2 DR15 DR7	A3 A11 B5 B51 B35 DR4	yes	yes	
Y22	A2 A30 A19 B13 B62 B15 DR13 DR6 DR7	A2 B8 B62 B15 DR17 DR3 DR13 DR6	A2 B15 B62 B40 B60 DR4 DR6 DR13	yes	yes	
Y23	A2 B8 B44 B12 DR15 DR2 DR13 DR6	A1 A2 B8 DR15 DR2 DR17 DR3	A2 A19 A32 B8 B12 B44 DR7	yes	yes	
Y24	A19 A30 A32 B5 B52 B13 DR2 DR15 DR7	A19 A29 B12 B44 B17 B58 DR7 DR8	A19 A32 A28 A68 B7 B40 B61 DR2 DR15 DR4	yes	yes	
Y25	A1 A34 B49 B57 DR3 DR9	A1 A68 B35 B57 DR1 DR9	A1 A19 A31 B8 B21 B50 DR3 DR17 DR7	no	no	recipient received T cell depletion

O= older recipient, Y= young recipient
don=donor response; 3P= third party response

Supplementary Table 2: Antibodies used in flow cytometry panels

Marker	Fluorochrome	Titer (µL)*		Company	Clone
		E	I		
**Phenotype & Cytokine Panel**					
CD137	APC	5	2	BD	4B4-1
CD3	AlexaFluor 700	0.25		BioLegend	HIT3a
CD4	PerCPCy5.5	1		BioLegend	RPA-T4
CD8	BV510	1		BioLegend	RPA-T8
CD45RA	BV785	1		BioLegend	HI100
CCR7	PE	1		BioLegend	G043H7
CD28	PE-CY7	0.5		BioLegend	CD28.2
IL2	BV421		0.5	BD	MQ-17H12
TNFα	BV605		1	BD	Mab11
IFNγ	BV711		0.5	BD	4SB3
**Proliferation Panel**					
CD3	BV510	1		BD	HIT3a
CD4	PerCP-Cy5.5	0.5		BioLegend	RPA-T4
CD8	APC-R700	1		BD	RPA-T8
CD45RA	BV650	0.25		BioLegend	HI100
CCR7	BV421	0.5		BioLegend	G043H7
CD28	BV785	1		BioLegend	CD28.2
CD27	BV605	2.5		BD	L128
**IL-2 receptor expression**					
CD25	APC	0.25		Biolegend	BC96
CD122	PE	2		BD	Mik-β2
CD132	BV421	2		BD	AG184
**All**					
CD14	APC-H7	0.125		BD	MφP9
CD19	APC-H7	0.5		BD	SJ25C1
CD56	APC-Cy7	0.5		BioLegend	HCD56
FVS780		0.25**		BD	none

* µL MoAb/50 µL cell suspension volume; **µL MoAb/ 1 mL cell suspension of max 10 million cells

E: extracellular

I: intracellular

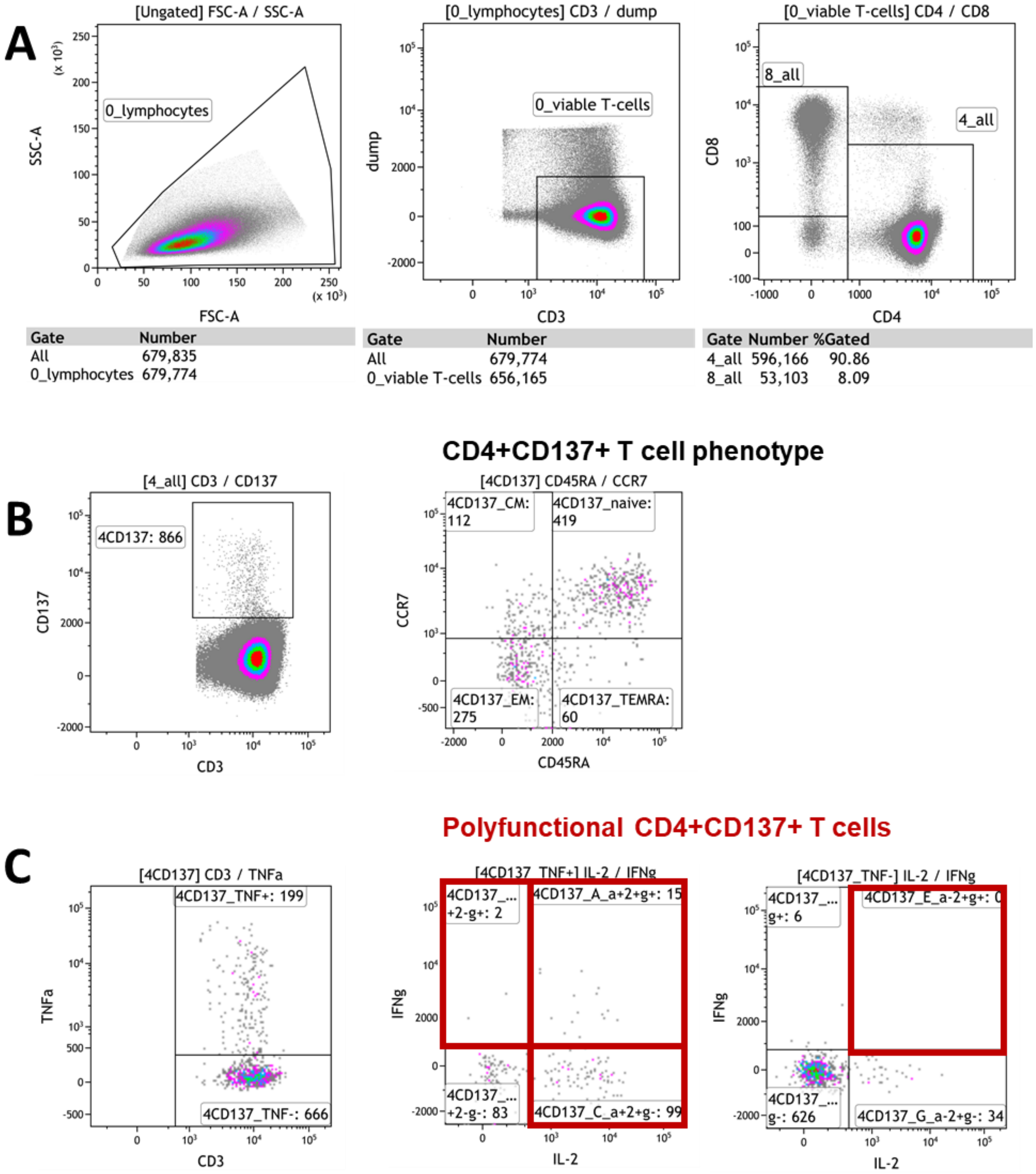

Supplementary Figure 1. Representative flow cytometry gating for characterizing phenotype and cytokine expression of alloreactive CD137+ CD4+ T cells. Gating strategy used to identify viable T lymphocytes (CD3+DUMP-), which were then divided into the CD4+and CD8+ T cell subset **(A)**. Alloreactive CD4+ T cells were identified by CD137-expression and then the phenotype was determined by dissecting these CD137+CD4+ T cells into the different T cell subsets, i.e. naïve, CM, EM and TEMRA, based on CCR7 and CD45RA expression **(B)**. Cytokine expression of alloreactive CD137+ CD4+ T cells was measured through first gating CD137+ CD4+ T cells according to TNFα expression (left plot). The TNFα positive (middle plot) and negative (right plot) fractions were further gated according to IFNγ and IL2 expression. For determining percentages of polyfunctional CD137+CD4+ T cells, gates (indicated in red) positive for two (IFNγ+TNFα-IL2+, IFNγ+TNFα+IL2-, IFNγ-TNFα+IL2+) or all three pro-inflammatory cytokines (IFNγ+IL2+TNFα+) were combined using a Boolean gate (**C**). A similar approach was used for alloreactive CD137+ CD8+ T cells.

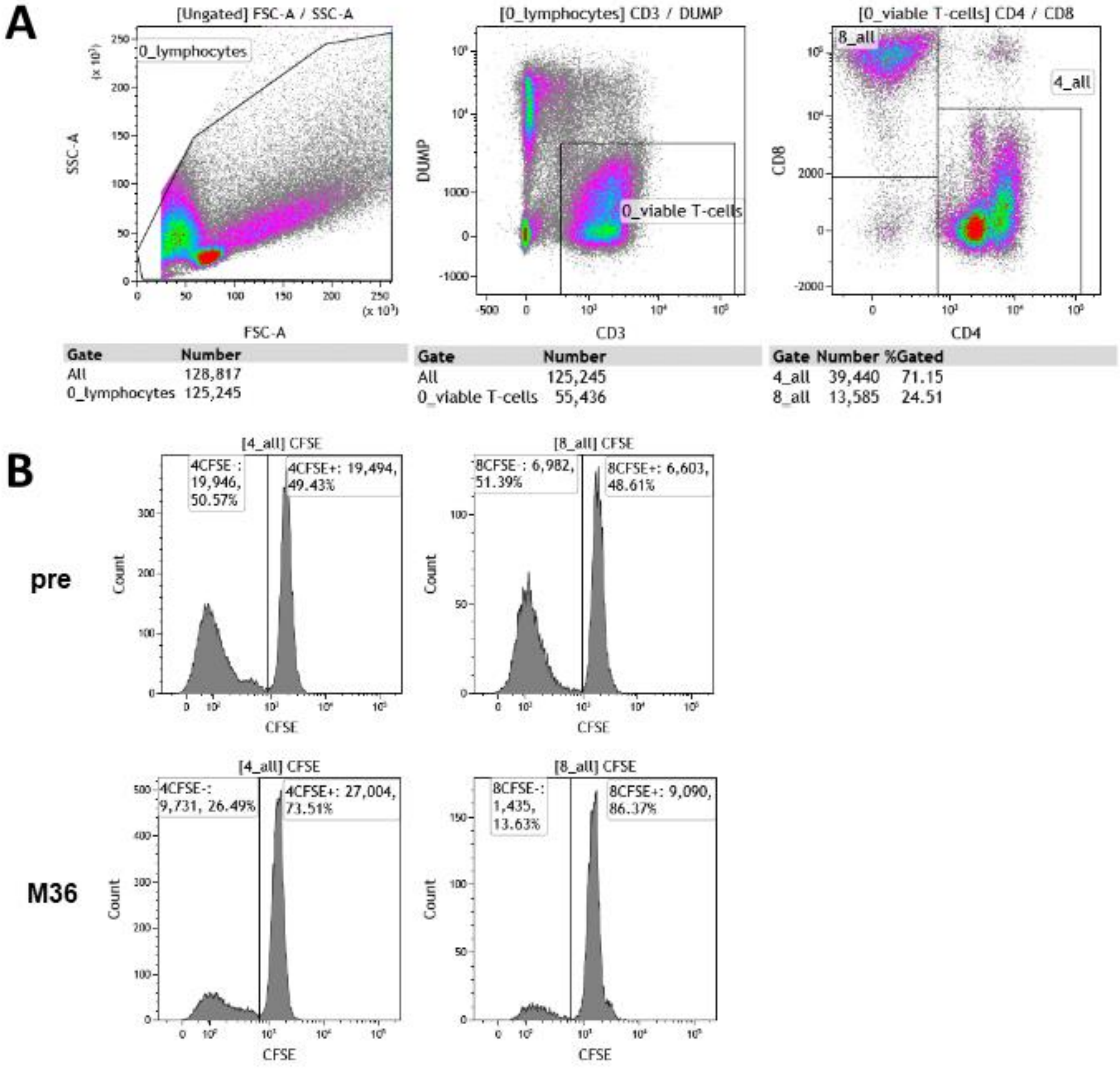

Supplementary Figure 2. Representative flow cytometry gating for measuring proliferating donor-reactive CD4+ and CD8+ T cells. Gating strategy used to identify viable T lymphocytes (CD3+DUMP-), which were then divided into the CD4+and CD8+ T cell subset (**A**). Representative gating of proliferating (CFSE-negative) CD4+ (left) and CD8+ (right) T cells from pre-transplant (top) compared to 36 months post-transplant (bottom) in a donor-stimulated sample (**B**).

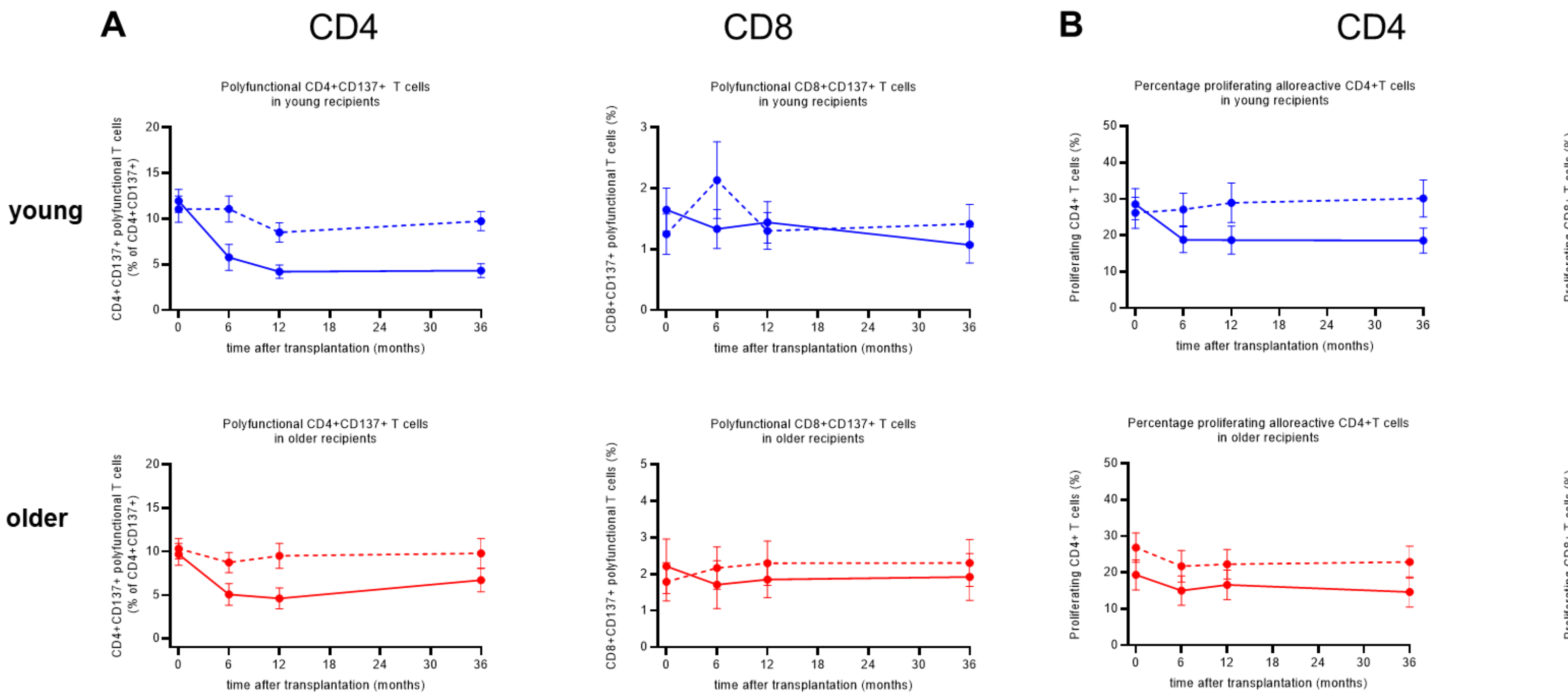

Supplementary Figure 3. Percentages of polyfunctional and proliferating alloreactive T cells in response to donor or third-party antigen post-transplant in stable kidney transplant recipients. Comparison of polyfunctional CD137+CD4+ (left) and CD137+CD8+ (right) T cell percentages (**A**) as well as dividing CD4+ (left) and CD8+ (right) T cell percentages (**B**) between donor-stimulated (solid line) and third-party stimulated (dotted line) samples in young (top, blue) and older (bottom, red) kidney transplant recipients.

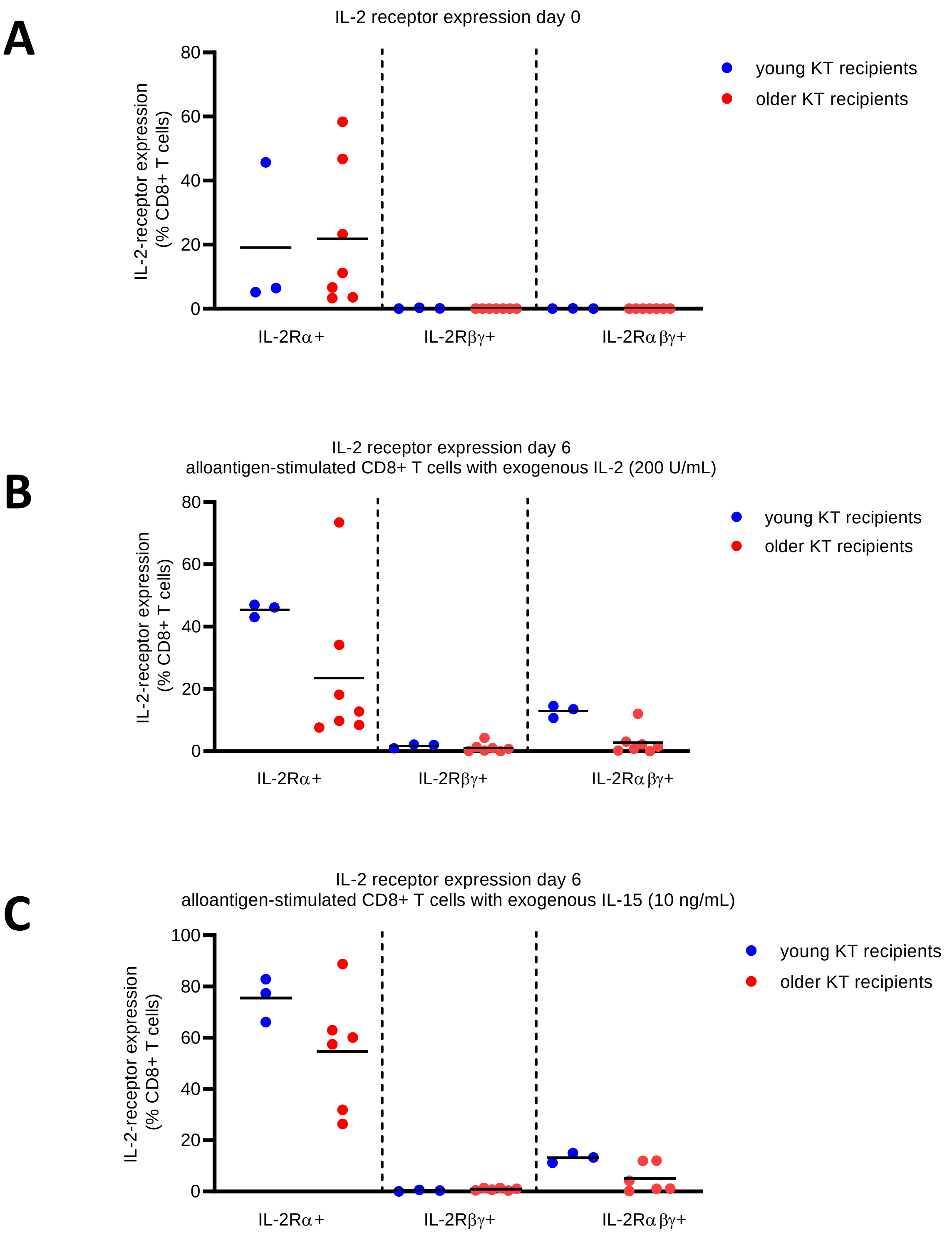

Supplementary Figure 4 IL2 receptor expression CD8+ T cells

Expression of low (IL2Rα), intermediate (IL2Rβγ) and high affinity (IL2Rαβγ) IL2 receptor was measured at day 0 **(A)** and day 6 following alloantigen-stimulation in presence of 200 U/mL IL2 (**B**) and 10 ng/mL IL15 (**C**), respectively. Blue circles represent young kidney transplant recipients and red circles represents older kidney transplant recipients. The horizontal line depicts the mean % of IL2R-expressing CD8+ T cells.

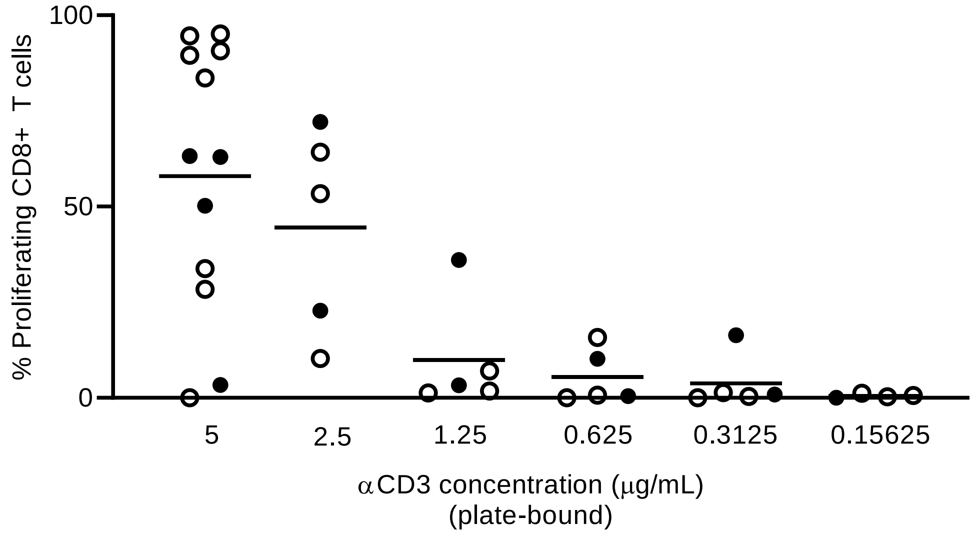

Supplementary Figure 5 Dose response of plate-bound anti-CD3 induced proliferation of isolated CD8+ T cells

Isolated CFSE-labelled CD8+ T cells were stimulated with different doses of plate-bound anti-CD3 for 6 days after which the cells were harvested and proliferation was measured using CFSE-dilution by flowcytometry. Open symbols represent the older KT recipients and the closed symbols the young KT recipients. The horizontal line depicts the mean % of proliferation.
